# Analytical Modelling of a Refractive Index Sensor Based on an Intrinsic Micro Fabry-Perot Interferometer

**DOI:** 10.3390/s151026128

**Published:** 2015-10-15

**Authors:** Everardo Vargas-Rodriguez, Ana D. Guzman-Chavez, Martin Cano-Contreras, Eloisa Gallegos-Arellano, Daniel Jauregui-Vazquez, Juan C. Hernández-García, Julian M. Estudillo-Ayala, Roberto Rojas-Laguna

**Affiliations:** 1 Departamento de Estudios Multidisciplinarios, División de Ingenierías, Universidad de Guanajuato, Av. Universidad s/n, Col. Yacatitas, Yuriria, Gto., C.P. 38940, Mexico; E-Mails: evr@ugto.mx (E.V.-R.); mcano_cco@utsoe.edu.mx (M.C.-C.); egallegos@ugto.mx (E.G.-A.); 2 Departamento de Tecnologías de la Información y Comunicación, Universidad Tecnológica del Suroeste de Guanajuato, Carretera Valle-Huanímaro km. 1.2, Valle de Santiago, Gto., C.P. 38400, Mexico; 3 Departamento de Mecatrónica, Universidad Tecnológica de Salamanca, Av. Universidad Tecnológica #200, Col. Ciudad Bajío, Salamanca, Gto., C.P. 36766, Mexico; 4 Departamento de Electrónica, División de Ingenierías, Universidad de Guanajuato, Carretera Salamanca-Valle de Santiago km 3.5 + 1.8, Comunidad de Palo Blanco, Salamanca, Gto., C.P. 36885, Mexico; E-Mails: jaureguid@ugto.mx (D.J.-V.); jchernandez@ugto.mx (J.C.H.-G.); julian@ugto.mx (J.M.E.-A.); rlaguna@ugto.mx (R.R.-L.)

**Keywords:** Fabry-Perot interferometer, fiber optics sensors, interferometry, non dispersive sensing, tunable laser spectroscopy

## Abstract

In this work a refractive index sensor based on a combination of the non-dispersive sensing (NDS) and the Tunable Laser Spectroscopy (TLS) principles is presented. Here, in order to have one reference and one measurement channel a single-beam dual-path configuration is used for implementing the NDS principle. These channels are monitored with a couple of identical optical detectors which are correlated to calculate the overall sensor response, called here the depth of modulation. It is shown that this is useful to minimize drifting errors due to source power variations. Furthermore, a comprehensive analysis of a refractive index sensing setup, based on an intrinsic micro Fabry-Perot Interferometer (FPI) is described. Here, the changes over the FPI pattern as the exit refractive index is varied are analytically modelled by using the characteristic matrix method. Additionally, our simulated results are supported by experimental measurements which are also provided. Finally it is shown that by using this principle a simple refractive index sensor with a resolution in the order of 2.15 × 10^−4^ RIU can be implemented by using a couple of standard and low cost photodetectors.

## 1. Introduction

In recent years fiber optic refractive index (RI) sensors have gain attention since these have interesting characteristics which allow them to be used in a broad range of chemical and biological sensing applications [1,2]. Moreover, fiber optic RI sensors can be implemented in different ways, such as by using fiber Bragg gratings [3,4,5,6], long period gratings [7,8,9], interferometers [10,11,12], resonators [13,14,15,16] and tapers [2,9,17,18]. Among these the Fabry-Perot interferometer (FPI) is a very popular option since it can be fabricated in different ways [19,20,21,22,23,24,25,26,27,28,29,30,31,32,33,34,35]. For instance, FPIs can be implemented by forming an air cavity between two optical fiber segments placed within a microcapillary [19,20]. Another technique consists in forming an open micro-notch cavity by micromachining either a single mode or a photonic crystal fiber with a fs laser [21,22]. FPIs at the tip of an optical fiber have also been implemented by different fabrication processes [23,24,25,26,27,28,29]. As an example, the technique presented by [23] consisting in produce a circular hole at the center of the cross-section area of a single-mode fiber (SMF) by means of a laser micromachining system. Afterwards, this micro-machined segment of SMF is spliced to another segment of optical fiber in order to form an air cavity. Finally the spliced fiber is cleaved forming one of the FPI optical mirrors [23], the other mirror will be the core of the micromachined SMF itself. Another way to form an FPI at the tip of an optical fiber is by fabricating an air microbubble [25,26,27,28,29]. In these FPIs the air cavity is formed by splicing a segment of hollow core photonic crystal fiber (HCPCF) to one SMF segment. Afterwards the HCPCF is cleaved by applying electrical discharges with a fusion splicer. Here a bubble of air is trapped within the HCPCF forming the FPI air microcavity and the HCPCF material that gets fused, when the electrical discharges are applied, will form the exit FPI mirror. Fiber tip FPIs can be used for refractive index sensing since within the exit FPI mirror multiple internal reflections will occur, if it has flat and parallel surfaces [36]. These reflections generate a well defined FPI spectrum, which fringe contrast depends on the refractive index of the medium around the FPI sensor tip. Usually the spectral response of this FPIs is analytically modelled by calculating the reflection coefficients of the three reflecting surfaces [25,26,27,28,29,30] which have very low reflectivity mirrors. In this paper the spectral response of the microcavity FPI is modelled by using the characteristic matrix method. This analytical model is not limited to solve FPIs formed by low reflectivity mirrors and on the contrary it can be used to spectrally model similar fiber FPIs tips that can have longer optical thickness and mirrors with higher refractive indexes [33,34,35]. Moreover, in some cases the optical thickness of the mirrors and the numerical aperture of the fiber can induce effects on the spectral FPI fringe pattern, all these effects can be modelled by applying the characteristics matrix method. Furthermore, this mathematical model allowed us to characterize a refractive index sensor.

Besides the interferometric analysis and characterization of the FPI it is necessary to implement a sensor configuration that allows us to determine in a simple way the refractive index measurements. Therefore in this work it is proposed to implement a sensor setup based on a combination of the Non Dispersive Sensing (NDS) and the Tunable Laser Spectroscopy (TLS) principles. Here in order to have one reference and one measurement channels a single-beam dual-path configuration is used for implementing the NDS principle. These channels are monitored with a couple of identical optical detectors, which output signals are correlated to calculate the overall sensor response, called here the depth of modulation. It is shown that this is useful to minimize drifting errors due to source power variations. By using this configuration it is shown that a sensor with a sensitivity of 3.25 V/RIU and a resolution of 2.15 × 10^−4^ RIU, within the refractive index range from 1.3 to 1.7, can be implemented. Finally, experimental measurements for the FPI characterization and for the overall sensor response are provided to support our simulation results.

## 2. Refractive Index Sensing Setup

**Figure 1:**
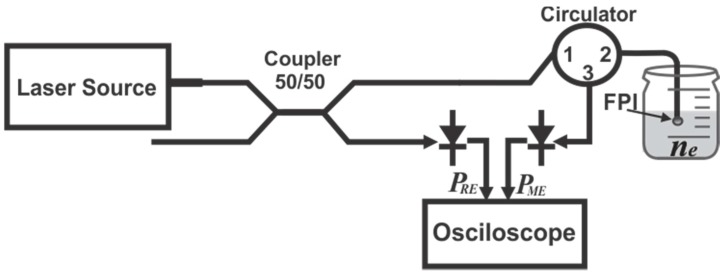
Refractive index sensing setup.

In this work a refractive index sensing setup based on a micro-FPI and on the non-dispersive sensing (NDS) principle of operation is presented. In general sensors based on NDS cannot resolve the spectrum of the sample, since all wavelengths provided by the source reach the detector at the same time and therefore it is neither possible to determine at which frequency a change in the spectrum occurs nor its intensity. However, these sensors can be implemented relatively easy, for instance the simplest NDS setup consists in passing a light beam through the analyte and the transmitted or reflected light is measured by an optical detector. Here the changes occurring in the analyte will induce a power variation which is observed by the detector. However due to the simplicity of this configuration it can be very sensitive to variations in the light source, inducing measurement errors. Therefore, there are several designs based on the NDS principle, but some of these configurations are designed to increase the sensitivity, the resolution and their ability to “identify” changes that are due only to the analyte and discard or compensate some other external changes, for instance variations in the light source. In the literature very different arrangements for different applications can be found, based on the NDS principle [37,38,39,40]. One possible way to classify these sensors is by the number of sources (beams), wavelength (filters), paths and detectors (channels) that they use. Some of the basic and simplest possible configurations are: single-beam single-wavelength [39], dual-beam single-wavelength [38], single-beam dual-wavelength [37], single-beam dual-path [40] and tunable laser spectroscopy (TLS) [41,42]. In our case we propose a refractive index sensor based on the NDS principle in which the single-beam dual-path and the TLS configurations are combined (Figure 1).

For this configuration only one laser source was used, which is divided into two paths by a 50/50 fiber coupler. One of these paths will be our reference channel and the second one will be the measurement channel, which satisfies the single-beam and dual-path concept. Moreover, the source is a laser emitting at 1550 nm and it can be tuned to a few nanometers, which is the basis of the TLS. Moreover, the beam on the measurement channel travels toward the FPI through the circulator (6015-3, Thorlabsv, Newton, NJ, USA) from the port 1 to the port 2. Afterwards, the reflected power $\left(P_{ME}\right)$ is detected by the measurement channel detector, while the reference channel power $\left(P_{RE}\right)$ is monitored by the reference detector. In this sensing arrangement the FPI spectrum is formed by a periodic fringe pattern which contrast will depends on the refractive index of the exit medium. Therefore the measurement channel detector will observe changes depending on the refractive index of the external medium $\left(n_{e}\right)$ while the reference channel detector will observe ideally a constant signal. These monitored signals will be correlated in order to obtain the depth of modulation, which can be useful to reduce drifting errors due to variations on the laser source. Hence in next section we will analyse and characterize the spectral response of the FPI as a function of the exit refractive index and later the overall output of our NDS arrangement.

## 3. Characterization of the Micro FPI

**Figure 2:**
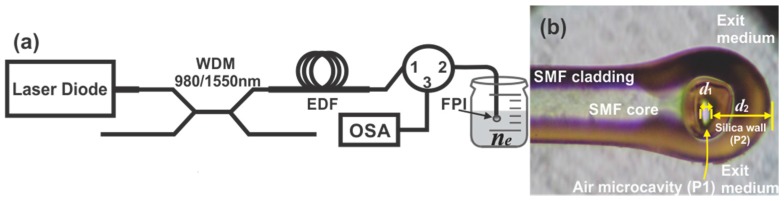
(**a**) Setup used to characterize the micro FPI spectral response as a function of the exit medium refractive index; (**b**) Picture of the fabricated FPI.

In order to characterize the spectral response of the FPI as a function of the exit refractive index medium the setup shown in Figure 2a was implemented. Here, the light of a pigtailed diode laser emitting at λ= 980 nm, delivering a maximum output power of 200 mW, was coupled to a wavelength division multiplexer (QFBGLD-980-200, Qphotonics, Ann Arbor, MI, USA) to pump an erbium doped fiber (F-EDF-T3, Newport, Newport Corporation, Irvine, CA, USA) of 3.4 m length. Afterwards, the luminescence generated by the EDF travels toward the FPI through the circulator (Thorlabs 6015-3) from the port 1 to the port 2. Finally, the reflected interference spectrum of the FPI was monitored at the port 3 of the circulator by using an optical spectrum analyser (OSA, AQ6370C, Yokogawa, Musashino-shi, Tokyo, Japan) with a resolution of 0.02 nm. In order to measure refractive index changes, the FPI was placed into a cuvette which was filled with different liquids that have calibrated refractive indexes. In this arrangement the sensor head is the intrinsic FPI which is based on an air microcavity. This was fabricated by splicing a segment of hollow core photonic crystal fiber (HC-1064-19 Cells Fiber Crystal, Blokken, Birkerød, Denmark) to a standard single mode fiber (SMF). Here to splice both fibers a conventional arc fusion splicer (Fitel-S175) was used and the fabrication process described in detail by [28] was followed. In this process after fibres get spliced multiple electric discharges are applied in order to cleave the photonic crystal fiber. After this step an air microcavity FPI is obtained at the tip of the SMF. A picture of the fabricated MFPI is shown in Figure 2b.

### FPI Principle of Operation

**Figure 3:**
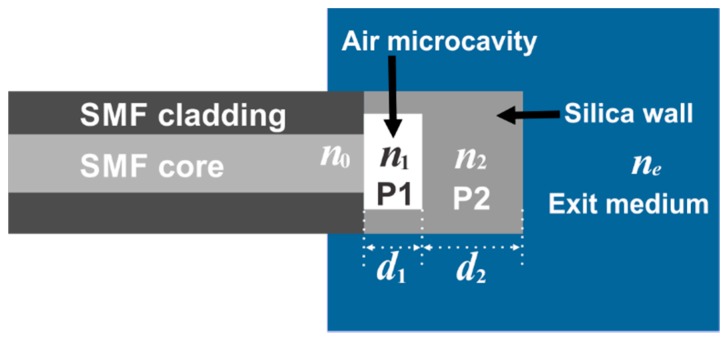
FPI structure.

The sensor head is basically a simple FPI which has the structure shown in Figure 3. Here the FPI mirrors are formed by the SMF core and by the thin silica wall at the tip of the fiber, which are separated by the air microcavity (Figure 3). In order to determine the overall reflectivity spectrum of the FPI structure we considered it as a pile of plane and parallel plates. We assumed this because our structure is a near planar FPI since the air microcavity length is much shorter than the radius of curvature of the exit mirror (*d*_1_ << *r*) (Figure 2b) [43,44,45,46]. For the case of near planar FPIs the finesse and the maximum transmission will be affected due to parabolic errors induced by mirrors curvature [45,46]. These mirror parallelism errors will strongly affect the FPI spectrum particularly for high reflectivity values [45]. Hence as we have a near planar FPI with very low reflectance mirror (≈0.04) therefore can be expected that the parabolic errors will not strongly affect the FPI spectral response. Consequently the structure can be assumed as a simple FPI formed by two flat plates. The first plate (P1) is the air microcavity with length $d_{1}$, while the second plate (P2) is the thin silica wall at the tip of the FPI with thickness $d_{2}$. Finally the incident medium of the pile of plates is the SMF core, with a refractive index $n_{0}$, and the exit medium, which is surrounding the FPI tip, has a refractive index $n_{e}$.

Hence, as the overall FPI is seen as a pile of plates therefore the final FPI reflectivity fringe pattern can be calculated by using the characteristic matrix. By using this method all reflections occurring at each interface of the FPI are taken into account which allow us to simulate with higher precision the reflectivity spectrum. The characteristic matrix of the whole medium can be expressed as [47]:

(1) $$\text{M}=\prod_{j=1}^{N}{\text{M}}_{j}=\left[\begin{array}{cc} a & ib\\ ic & d \end{array}\right]$$

where ${\text{M}}_{j}$ is the characteristic matrix of the *j*-th plate, which for an unpolarised beam is given by [48]:

(2) $${\text{M}}_{j}=\left[\begin{array}{cc} \cos\left(\frac{2\text{π}n_{j}d_{j}\cos{\text{θ}}_{j}}{\text{λ}}\right) & -\frac{i}{n_{j}}\sin\left(\frac{2\text{π}n_{j}d_{j}\cos{\text{θ}}_{j}}{\text{λ}}\right)\\ -in_{j}\sin\left(\frac{2\text{π}n_{j}d_{j}\cos{\text{θ}}_{j}}{\text{λ}}\right) & \cos\left(\frac{2\text{π}n_{j}d_{j}\cos{\text{θ}}_{j}}{\text{λ}}\right) \end{array}\right]$$

where $n_{j}$, $d_{j}$, ${\text{θ}}_{j}$ are the refractive index, the thickness, and the incident angle of the *j*-th plate respectively and λ is the wavelength. The reflectance of this plate assembly can be obtained by using the following definition [48]:

(3) $$\left[\begin{array}{c} B\\ C \end{array}\right]=\left[\begin{array}{cc} a & ib\\ ic & d \end{array}\right]\left[\begin{array}{c} 1\\ n_{e} \end{array}\right]$$

Thus, the reflection coefficient is given by:

(4) $$r=\frac{n_{0}B-C}{n_{0}B+C}$$

and finally the reflectivity of the overall assembly is given by:

(5) $$R_{FP}\left(\text{λ},n_{e}\right)=\left(\frac{n_{0}B-C}{n_{0}B+C}\right){\left(\frac{n_{0}B-C}{n_{0}B+C}\right)}^{*}$$

**Figure 4:**
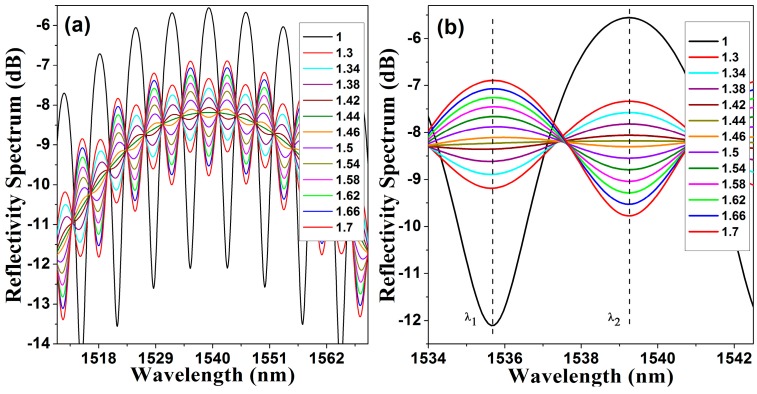
(**a**) Simulated FPI reflectivity spectra considering an exit medium with different refractive indexes; (**b**) Detail of the FPI reflectivity spectra showing a single fringe peak and valley.

Therefore in our system the refractive index $n_{0}=$ 1.44, the P1 has a $n_{1}=$ 1 and P2 has a $n_{2}=$ 1.44. Finally the exit medium *n_e_* will depend on the material surrounding the micro FPI structure (Figure 2). Simulated reflectivity spectra of the FPI for different *n_e_* are shown in Figure 4a. For these simulations we considered *d*_1_ = 12.7 μm and *d*_2_ = 110.9 µm. As it can be appreciated in this type of FPI neither the fringe positions nor the free spectral range will change as *n_e_* is varied [49,50,51]. Here only the contrast and the finesse of the spectral fringes occurring by multiple internal reflections within P2 will be modified (Figure 4b).

## 4. Experimental Results of the FPI Spectrum

**Figure 5:**
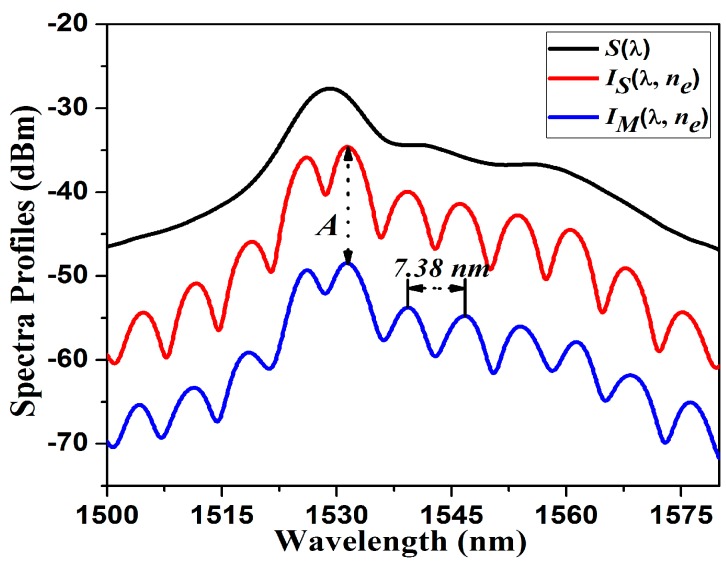
Measured source spectral profile S(λ); $I_{M}\left(\text{λ},n_{e}\right)$ and $I_{S}\left(\text{λ},n_{e}\right)$ are the measured and the simulated FPI reflection spectrum respectively. For this case $n_{e}=1$ was considered.

In Figure 5 *S*(λ) is shown, which is the spectral profile of the luminescence generated by the EDF, which illuminates the micro FPI. Moreover, in this figure it is also shown the measured reflection spectral profile $I_{M}\left(\text{λ},n_{e}\right)$ recorded with the OSA (Figure 2). From this measured spectral profile it is possible to determine the exact thickness of the thin silica wall (P2), where multiple internal reflections occur. Here it is important to recall that the Free Spectral Range (*FSR*), which is the separation between two consecutive fringes, is defined as:

(6) $$FSR=\frac{{\text{λ}}^{2}}{2n_{2}d_{2}}$$

**Figure 6:**
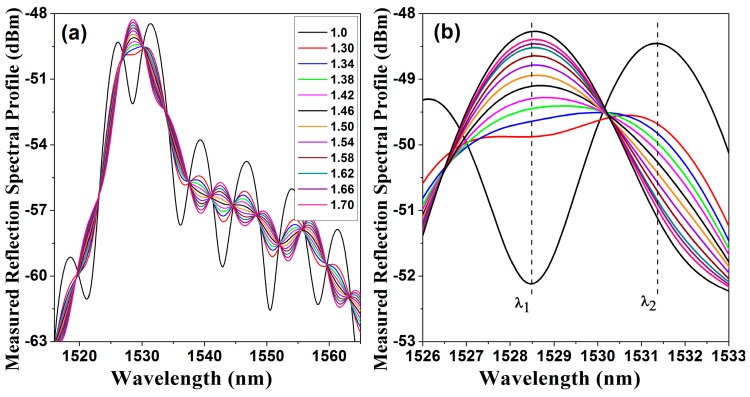
(**a**) Measured reflected power spectra $I_{M}\left(\text{λ},n_{e}\right)$ for different values of exit medium refractive index; (**b**) detail of the $I_{M}\left(\text{λ},n_{e}\right)$ spectra showing only a single spectral FPI fringe.

Therefore if the measured *FSR* = 7.38 nm nm then *d*_2_ = 110.9 µm, which is very close to the experimentally measured value. The measured air microcavity for this FPI was approximately *d*_1_ = 12.7 μm, this was measured by using an optical microscope. Additionally, in Figure 6 it is also shown the simulated spectral profile of the light reflected by the FPI, which is given by *I_S_*(λ,*n_e_*) = *S*(λ)*R_FP_*(λ,*n_e_*).

In our FPI insertion losses occurred, which are mainly due to the photonic crystal and single mode fibers splicing. These losses are basically flat over the spectrum (Figure 5) and can be described as *A* = *I_M_* − *I_S_* ≈ 13.85 dB. For this calculation we are taking as a reference the case when the exit medium is air $\left(n_{e}=1\right)$.

Additionally, the measured FPI reflection spectral profile is shown in Figure 6a. For this experiment the exit medium was changed in order to vary $n_{e}$. In Figure 6b a detail of the spectral profile is presented.

### Characterization of the Amplitude of the FPI Fringes as a Function of the Exit Medium Refractive Index

**Figure 7:**
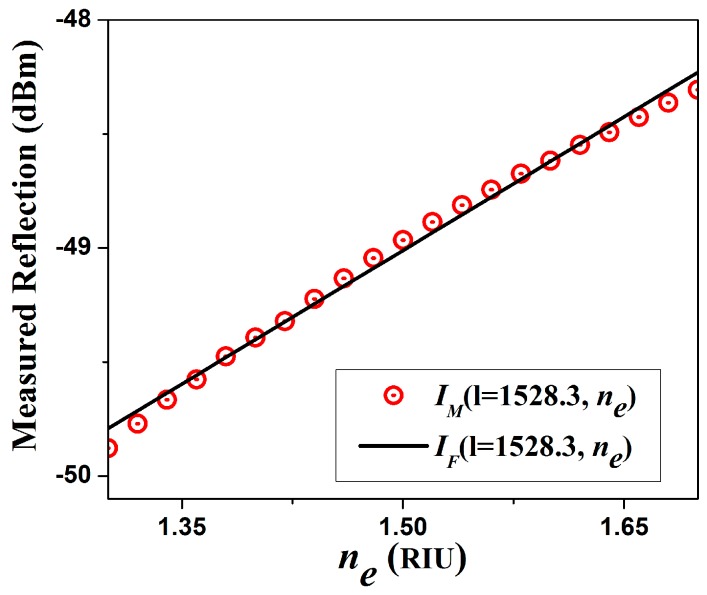
Measured reflected energy at λ=1528.3 nm as a function of the exit medium refractive index and its linear fitting.

As the contrast and the finesse of the spectral fringes change as the refractive index of the exit medium is varied, therefore it is possible to detect these changes by measuring the amount of reflected light at certain wavelength. This change will be not linear, since it depends on the reflectivity of the exit interface, formed by mirror (P2) and the exit medium however it is completely predictable. Hence for instance, by using the arrangement shown in Figure 2 the spectral response presented in Figure 6b was obtained. From these spectra the fringe that has the maximum reflection level is analysed, in our case this occurs at λ=1528.3 nm. Moreover, the reflected energy, in dBm, as a function of the exit medium refractive index, $I_{M}\left(1528.3,n_{e}\right)$, is presented in Figure 7. For this example, $I_{M}\left(1528.3,n_{e}\right)$ can be fitted very well by a linear function $I_{F}\left(1528.3,n_{e}\right)$, as follows:

(7) $$I_{F}\left(1528.3,n_{e}\right)=3.906n_{e}-54.87,\;\text{for}\;1.30\leq n_{e}\leq 1.70$$

This change in reflected power can be taken into advantage to implement a refractive index sensor. The main problem is that we will need a very narrow filter at λ=1528.3 nm in order to implement a practical sensor. Otherwise is to replace the broad band source by a laser that can be tuned near to the peak or valley of one of the FPI fringes. Here we proceed to implement a practical sensor based on a tuneable fiber laser.

## 5. Analysis of the Non Dispersive Refractive Index Sensor

A single-beam single-path ND sensor can be implemented in a relatively simple way, by replacing the broadband source by a single line laser. In general terms the expected sensor response will be described as shown in Figure 6. However this kind of sensor can be sensitive to drifting errors due to different factors such as instability in the laser line position, source power instability and aging of the source and detector. Hence the single-beam dual-path configuration (Figure 1) is useful to reduce this type of error. In this arrangement the energy reaching the measurement detector is directly described by the integrated optical FPI reflectance, which can be expressed as:

(8) $$P_{M}\left(n_{e}\right)=\int_{\text{λ}1}^{\text{λ}2}L\left(\text{λ}\right)R\left(\text{λ},n_{e}\right)d\text{λ}$$

where L(λ) is the laser emission profile and $R\left(\text{λ},n_{e}\right)$ is the FPI reflectance. The power of the reference channel can be described as:

(9) $$P_{R}=\int_{\text{λ}1}^{\text{λ}2}L\left(\text{λ}\right)d\text{λ}$$

**Figure 8:**
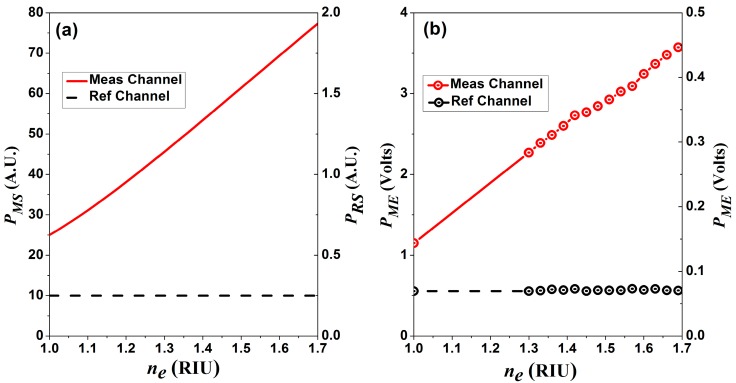
Detector outputs as a function of the exit refractive index, (**a**) simulated results; (**b**) experimental measurements.

In order to simulate the energy reaching both detectors L(λ) as was assumed as an ideal laser with Gaussian shape, central wavelength at 1550 nm, spectral width of 20 pm and normalized amplitude. The simulated overall output power of the measurement channel $P_{MS}\left(n_{e}\right)$ and of the reference channel output $P_{RS}$ are shown in Figure 8a. For this simulation the scale of the reference and the measurement channels are different since we are considering that both signals are amplified with different gain factors. This is because the measurement channel will have much more losses that the reference channel.

In our experiment a couple of model DET01CFC InGaAs detectors from Thorlabs Inc. were used, which have a noise equivalent power (*NEP*) of 1.5 × 10^−15^ W/$\sqrt{\text{Hz}}$, Responsivity (G) of approximately 1 A/W, dark current $\left(I_{Dark}\right)$ of 700 pA @ 1550 nm and the minimum load resistance $\left(R_{L}\right)$ allowed is 50 Ω. For these detectors the generated photocurrent $\left(I_{PD}\right)$ is defined as the product of the incident power (P) times the responsivity *I_PD_ = PG*. Moreover the output in terms of volts is given as $Vo=\left(I_{PD}+I_{Dark}\right)R_{L}$. Here it is important to recall that the NEP expresses the rms incident radiant power per square root bandwidth necessary to produce a signal to noise ratio of one [52]. The optical detector noise can be generated by different sources, either internal or external. For instance, some internal noise sources are Johnson, shot, 1/f, and generation recombination, and some external sources are photon flux, the interface electronics and microphonic noise [52]. Therefore, to have a S/N=1 it is necessary that P=NEP, this considering that Δf=1. Consequently the minimum detectable power that can be detected must be equal to 1.5 × 10^−15^ W, which will produce a minimum output voltage described by:

(10) $$Vo_{min}=\left(1.5\times 10^{-15}\text{W}\times 1\frac{\text{A}}{\text{W}}+700\times 10^{-12}\text{A}\right)R_{L}$$

In our case we used a $R_{L}=$ 50 Ω for the reference channel and 1 MΩ for the measurement channel. Hence, for the measurement channel $Vo_{min}\approx 7\times 10^{-4}$ V within 1.3 $\leq n_{e}\leq$ 1.7. Now, the experimentally measured voltage readings for each channel at different exit refractive index are shown in Figure 8b. From these measurements it is possible to determine that the sensor has a sensitivity of 3.25 V/RIU. Consequently, the resolution of the sensor will be in the order of 2.15 × 10^−4^ RIU. From Figure 8 it is clear that the tendency of the measured voltages is in general similar to the predicted by the simulation. However for this sensor if for some reason there is a change in the source characteristics it will induce an error in the final sensor response. For instance the sensor itself is expected to have more tolerance to small variation in the laser central wavelength since the spectral *FSR* is relatively large and the fringe contrast is very small. However changes in the power source can induce more significant measurement errors. For this reason some authors have proposed to correlate the reference and the measurement channels to calculate the depth of modulation which can useful to reduce this type of error.

### Depth of Modulation

In the sensing setup any variation occurring at the source will affect in the same proportion both channels, therefore by correlating both signals it is possible to minimize effects in the sensor response. Therefore instead to measure the sensor response directly from the measurement detector, we prefer to calculate the depth of modulation (*DM*) [40]. This is defined as the difference between the detected channels signals, divided by the mean of both signals [38] and can be written as:

(11) $$DM\left(n_{e}\right)=2\frac{P_{M}\left(n_{e}\right)-kP_{R}}{P_{M}\left(n_{e}\right)+kP_{R}}$$

**Figure 9:**
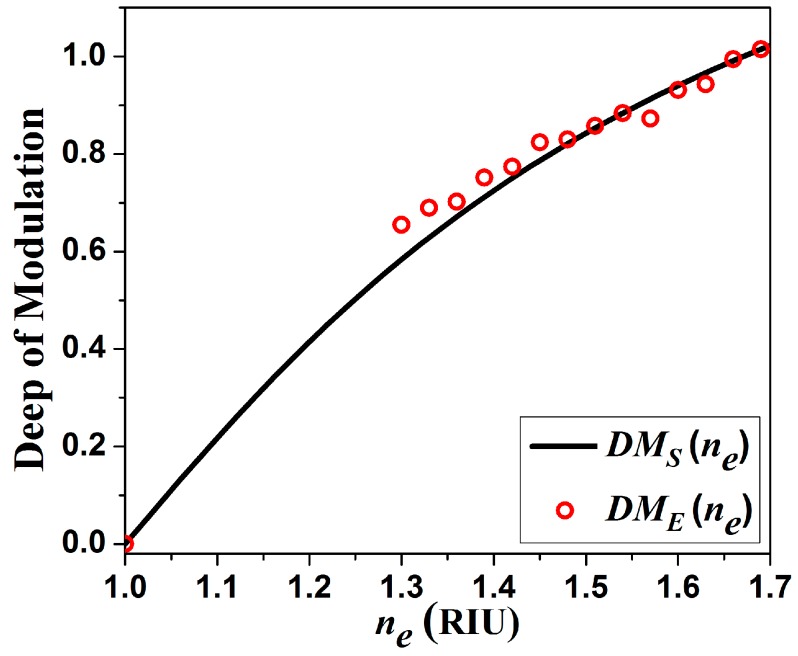
Calculated depth of modulation from simulated and measured signals.

In this case k is a proportionality constant to take into account the beam attenuation in the measurement channel, which mainly occur due to the FPI as was previously explained. In this way the $DM\left(n_{e}\right)$ gets normalized and unitless. The proportionality constant for our simulated signals (Figure 9a) is given as $k_{S}=P_{MS}\left(n_{e}=1.0\right)/P_{RS}$. Moreover for the measured channel the proportionality constant was determined as $k_{E}=P_{ME}\left(n_{e}=1.0\right)/P_{RE}$. In this way the depth of modulation calculated from simulated and from experimental measurements are presented in Figure 9. Here can be appreciated that the depth of modulation from experimental measurements fits well with our simulation. Now considering that we can detect changes in the RIU with a resolution of 2.15 × 10^−4^ therefore the valid change in the depth of modulation will be of approximately $\Delta D_{m}=$ 1.94 × 10^−4^. The main advantage of this sensing setup is that it compensate variations in the source. Moreover, the depth of modulation calculation can be performed rapidly by a very simple electronic stage based on a low cost microcontroller. Also within this electronic stage can be programmed the equation to convert the depth of modulation into RIU and display the final result on a screen.

## 6. Conclusions

In this work a comprehensive analysis and characterization of a non-dispersive refractive index sensor, based on an intrinsic FPI was presented. Here the FPI characterization was carried out by considering it as pile of plates and therefore its spectral response is modelled by the characteristic matrix method. Moreover it was shown that in this FPI, multiple internal reflections occur within the exit plate (P2). This effect allows the generation of a FPI fringe pattern, which is sensitive to changes on the refractive index of the exit medium. Due to the changes in the refractive index the fringe pattern contrast and finesse are affected. Therefore to calculate the fringe contrast of spectrum generated at the exit plate (P2) it was considered as a single unbalanced FPI. Here by using the proposed model the contrast and the overall reflection spectrum can be easily calculated. The overall sensor is based on the non- dispersive principle with a single-beam dual-path configuration combined with the tunable laser spectroscopy technique. Here by using standard and low cost detectors the resolution achieved is on the order of 2.15 × 10^−4^ RIU. The provided simulation results agree very well with the results obtained experimentally.
